# Exploring genetic confounding of the associations between screen time and depressive symptoms in adolescence and early adulthood

**DOI:** 10.1093/ije/dyag079

**Published:** 2026-06-11

**Authors:** Jiayao Xu, Jessie Baldwin, Amanda M Hughes, Annie Herbert, Hannah J Jones, Marcus R Munafo, Laura D Howe

**Affiliations:** MRC Integrative Epidemiology Unit, Bristol Medical School, University of Bristol, Bristol, BS1 5DS, United Kingdom; Population Health Sciences, Bristol Medical School, University of Bristol, Bristol, BS1 5DS, United Kingdom; Department of Clinical, Educational and Health Psychology, Division of Psychology and Language Sciences, University College London, London, WC1H 0AP, United Kingdom; Social, Genetic and Developmental Psychiatry Centre, Institute of Psychiatry, Psychology and Neuroscience, King’s College London, London, SE5 8AF, United Kingdom; MRC Integrative Epidemiology Unit, Bristol Medical School, University of Bristol, Bristol, BS1 5DS, United Kingdom; Population Health Sciences, Bristol Medical School, University of Bristol, Bristol, BS1 5DS, United Kingdom; MRC Integrative Epidemiology Unit, Bristol Medical School, University of Bristol, Bristol, BS1 5DS, United Kingdom; Population Health Sciences, Bristol Medical School, University of Bristol, Bristol, BS1 5DS, United Kingdom; MRC Integrative Epidemiology Unit, Bristol Medical School, University of Bristol, Bristol, BS1 5DS, United Kingdom; Population Health Sciences, Bristol Medical School, University of Bristol, Bristol, BS1 5DS, United Kingdom; National Institute for Health Research Bristol Biomedical Research Centre, University Hospitals Bristol NHS Foundation Trust, University of Bristol, Bristol, BS1 5DS, United Kingdom; Vice Chancellor’s Office, University of Bath, Bath, BA2 7AY, United Kingdom; MRC Integrative Epidemiology Unit, Bristol Medical School, University of Bristol, Bristol, BS1 5DS, United Kingdom; Population Health Sciences, Bristol Medical School, University of Bristol, Bristol, BS1 5DS, United Kingdom

**Keywords:** screen time, depression, genetic confounding, ALSPAC

## Abstract

**Background:**

Digital devices have become a major aspect of children’s lives. Associations between screen time and mental health have been observed, but the causality remains unclear. This study aimed to investigate the associations between screen time and later depressive symptoms, and to test the robustness of these associations when accounting for genetic confounding.

**Methods:**

This study used data from the Avon Longitudinal Study of Parents and Children—a prospective cohort of children born between 1991 and 1992 in the UK. Different forms of screen time and depressive symptoms at ages 16, 22, and 26 years were assessed through self-completion questionnaires. The average daily screen time was calculated. Depressive symptoms were measured by using the Short Mood and Feelings Questionnaire (SMFQ). Polygenic scores (PGSs) for depression were calculated. Linear regression models were used to examine the associations between standardized screen time at ages 16, 22, and 26 years, and standardized depressive symptoms at age 26 years, adjusting for sociodemographic confounders and PGSs. Genetic sensitivity analysis (Gsens) was used to test for genetic confounding in these associations.

**Results:**

A total of 3003 participants were included in the analysis. Some, but not all, forms of screen time were associated with higher SMFQ scores, e.g. time spent using a phone, tablet, or e-book at age 22 years [β: 0.10, 95% confidence interval (CI) 0.07, 0.14 for weekdays; β: 0.08, 95% CI 0.04, 0.11 for weekends] and television time at age 26 years (β: 0.10, 95% CI 0.06, 0.14 for weekdays; β: 0.09, 95% CI 0.06, 0.13 for weekends). These associations persisted after adjustment for sociodemographic confounders and PGSs, but were attenuated in the Gsens (β = 0.03, 95% CI −0.01, 0.07 for the association with time spent using a phone, tablet, or e-book at age 22 years on weekends; β = 0.06, 95% CI 0.01, 0.10 for television time at age 26 years on weekends).

**Conclusion:**

For some measures of screen time, there were no associations with depressive symptoms. Where associations were seen, they were attenuated in the Gsens, implying that genetic confounding is present in the relationship between screen time and depressive symptoms in adolescents and young adults.

Key Messages

- This study investigated the associations between screen time and later depressive symptoms during adolescence and early adulthood by using a range of approaches to examine confounding, including a genetic sensitivity model.
- Genetic confounding plays a role in the relationship between different types of screen time and depressive symptoms in adolescents and young adults, with the relationship between excessive screen time and depressive symptoms appearing weaker after accounting for genetic confounding.
- This study suggests that genetic confounding is present in the relationship between screen time and depressive symptoms in adolescents and young adults, highlighting the need to consider genetic factors when interpreting this association.

## Introduction

Digital technology is integral to children’s lives [1, 2], yet excessive use of digital devices is a global concern [3] and associated with negative health outcomes, including mental health problems such as depression in youth [3, 4]. Several national organizations recommend that both adolescents and young adults should limit their recreational screen time [5–7], although many young people exceed the recommended amount [2, 8].

Cross-sectional and longitudinal studies have indicated that excessive screen time is associated with increased depression [8, 9]. A meta-analysis of observational studies reported dose–response associations between screen time and risk of depression in children and adolescents [10]. However, genome-wide association studies (GWASs) have indicated high genetic correlations between leisure screen time and depression [11]. Young people with a higher genetic vulnerability to depression problems are more likely to engage in screen time based on the model of compensatory internet use (that people experiencing negative moods might turn to the digital world as an escape from real life [12]), as well as be more prone to developing depressive symptoms. Therefore, the associations between screen time and depressive symptoms could be inflated by genetic confounding.

A polygenic score (PGS) represents an estimation of an individual’s genetic predisposition towards a specific trait [13, 14]. Controlling for a PGS for depression helps account for potential genetic confounding when examining the associations between screen time and depression. However, PGSs only capture a small proportion of heritability in depression, and thus do not fully account for genetic confounding [15, 16]. This limitation can be addressed by conducting a genetic sensitivity analysis (Gsens) that estimates genetic confounding under scenarios in which the PGS captures additional genetic variance in depression. Specifically, this is achieved by deriving a latent PGS that captures SNP (single-nucleotide polymorphism)-based heritability in depression and controlling for this PGS when testing the associations between screen time and depression (for more details, see [17, 18] and “Methods”).

Two previous studies have applied this Gsens to investigate genetic confounding of the relationship between media use and mental health. First, using a cross-sectional study design with a sample of British twins assessed at age 22 years, Ayorech *et al.* [19] found that the associations between media use and mental health were explained largely by genetic influences, with these findings supported by bivariate twin analyses. Second, Zhang and colleagues [20] examined the association between screen time and parent-reported psychiatric problems a year later among US children from the Adolescent Brain Cognitive Development study and observed substantial genetic confounding. While these studies provide initial evidence to suggest genetic confounding, it is important to (i) test whether the findings are replicated in divergent samples with different measures of screen use and (ii) understand the extent to which genetic confounding affects longitudinal relationships between screen time and mental health, assessed several years later.

In this study, we addressed these research gaps by exploring genetic confounding in the associations between screen time and self-reported depressive symptoms by using three waves of longitudinal data collected during adolescence and early adulthood. We aimed to investigate the associations between screen time and later self-reported depressive symptoms and to test the robustness of these associations when accounting for genetic confounding using three waves of data in adolescence and early adulthood from a UK birth cohort study.

## Methods

### Dataset

The Avon Longitudinal Study of Parents and Children (ALSPAC) is a longitudinal study that recruited ∼15 000 mothers with expected dates of delivery between 1991 and 1992 in the former county of Avon, UK [21–25]. The initial number of pregnancies enrolled was 14 541, with 13 988 children alive at 1 year of age. The total sample size for analyses was 15 447 pregnancies and 14 901 children were alive at 1 year of age. For details, please refer to Supplementary Section 1.

### Inclusion and exclusion criteria

**Figure 1:**
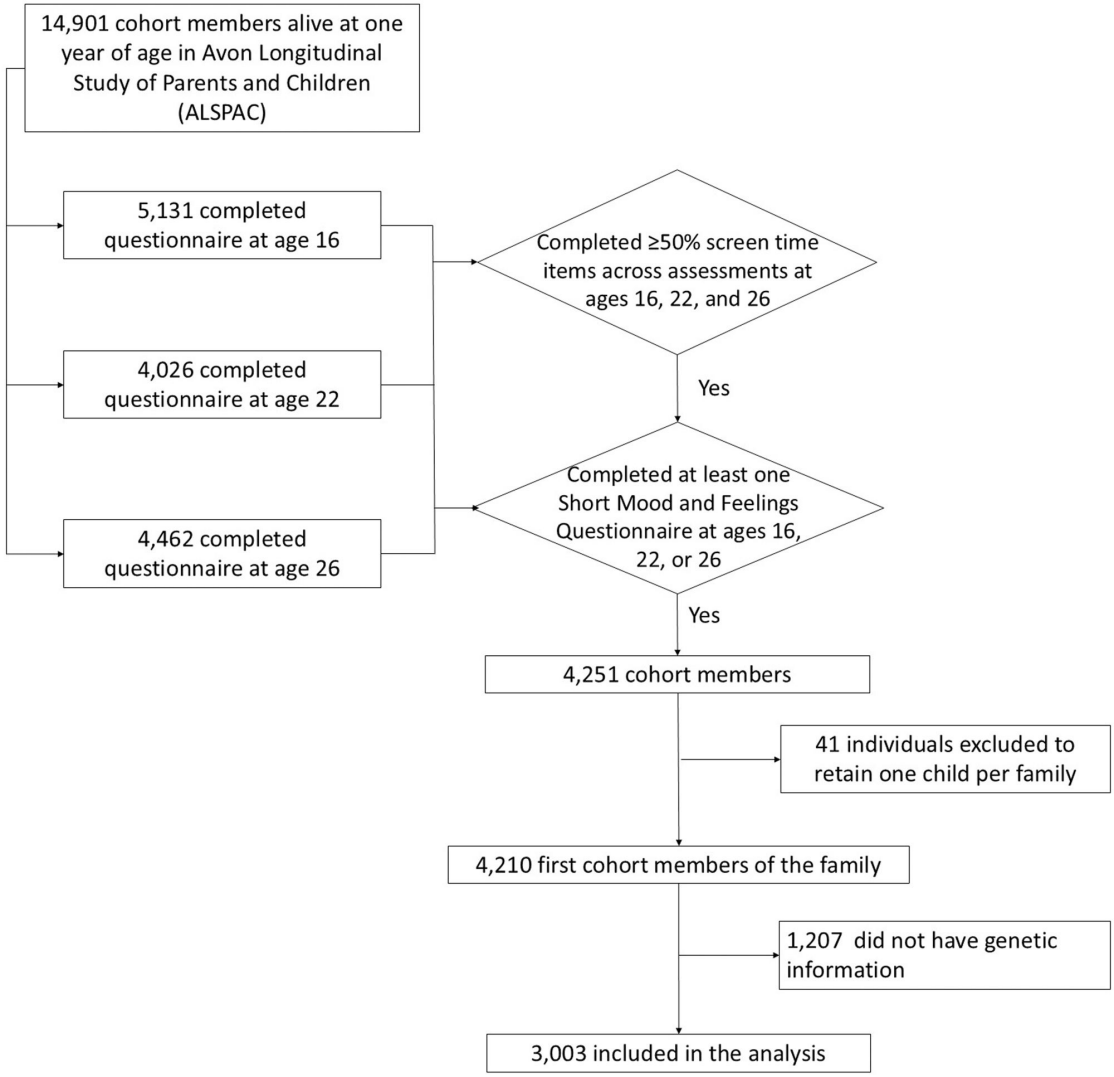
Flow diagram of the participants included in the analyses.

We included ALSPAC children who were alive at 1 year of age, responded to ≥50% of the screen-time questions across the assessments at ages 16, 22, and 26 years, and completed at least one questionnaire on depressive symptoms at ages 16, 22, or 26 years (Supplementary Table 1). For the 41 families with twins, we included the child who was born first. Participants without genotype data that passed quality control were excluded (Fig. 1).

### Measurement

#### Screen time

Participants’ self-reported screen-time use was collected at three time points through a self-completion questionnaire during adolescence and early adulthood: at ages 16, 22, and 26 years. They were asked questions about screen time separately for a normal weekday and a weekend day. At age 16 years, they were asked about the amount of time spent watching television, using a computer, texting, and talking on a mobile phone. At ages 22 and 26 years, they were asked about the amount of time spent watching television, playing video games on computers/laptops or game consoles, using a computer/laptop for purposes other than gaming, and using a phone, tablet, or e-book. Each response option was transformed into an ordinal variable by substituting it with the median of the corresponding category (e.g. “less than 1 hour” = 0.5) (further details of derivation and definitions are in Supplementary Table 2). The average daily screen time at each age was calculated by using the formula: (total screen time on weekdays × 5) +( total screen time on weekends × 2)/7 and then standardized for comparison across the three waves.

#### Depressive symptoms

Depressive symptoms were assessed by using the 13-item Short Mood and Feelings Questionnaire (SMFQ) at ages 16, 22, and 26 years [26]. The total score ranged from 0 to 26, where higher scores suggest more depressive symptoms. The SMFQ was reported by participants themselves at ages 16, 22, and 26 years (Supplementary Table 3). The SMFQ has previously demonstrated good validity and reliability within the ALSPAC cohort, with strict measurement invariance and Cronbach’s α ranging from 0.87 to 0.92 between ages 14 and 26 years [27, 28]. The SMFQ scores were standardized.

#### Genotyping and PGSs

Detailed information about genotyping and quality control in the ALSPAC is provided elsewhere [29]. For the quality-control process, please refer to Supplementary Section 1. PGSs for depression were generated by using GWAS summary statistics [30]. PGSs were derived by using PRSice software [16, 31] including SNPs regardless of their significance (*P *= 1) [13, 16]. SNPs were clumped [with a linkage disequilibrium (LD) clumping distance of 10 000 kb and *R*^2^ = 0.01) so that the retained SNPs are largely independent and thus their effects can be summed [32, 33]. The alleles associated with the phenotype were summed and weighted by their effect sizes reported in the corresponding GWAS to compute the PGSs. To control for population stratification, we residualized PGSs for the first 10 principal components estimated from the genome-wide SNP data [34]. PGSs were standardized.

#### Sociodemographic factors

Sociodemographic factors were: participants’ sex at birth (females/males), parental marital status (married/remarried/widowed/single never married/divorced/legally separated), parental highest education levels [maternal and partner highest education qualifications (CSE/vocational/O-level/A-level/degree)], parental highest occupational social classes [I/II/III (non-manual)/III (manual)/IV/V], and not in education, employment, and training (NEET) status (yes/no) (Supplementary Table 4). The parental marital status during pregnancy was combined into married/remarried, never married, and divorced/legally separated/widowed. All factors, except NEET status, were reported in questionnaires completed by the child’s main caregiver (usually the mother) during pregnancy. The NEET status [35] was based on self-reported information at age 16 years.

### Missing data

To maximize the sample size and reduce selection bias due to attrition (Supplementary Table 5), we used multiple imputations to ascribe missing screen time, depressive symptoms, and sociodemographic data (Supplementary Table 6). Missing data were imputed by using the “mice” package in R version 3.6.1 under a missing-at-random assumption. Considering the sex differences in screen time and depressive symptoms and possible interactions [36], data for females and males were imputed separately [37]. Variables were imputed by using 50 imputations with 30 iterations, the imputation model included all the variables in the analytic model (exposures, outcomes, covariates), and additional auxiliary variables that predict missingness or make the missing-at-random assumption more plausible (Supplementary Table 7).

### Data analyses

**Figure 2:**
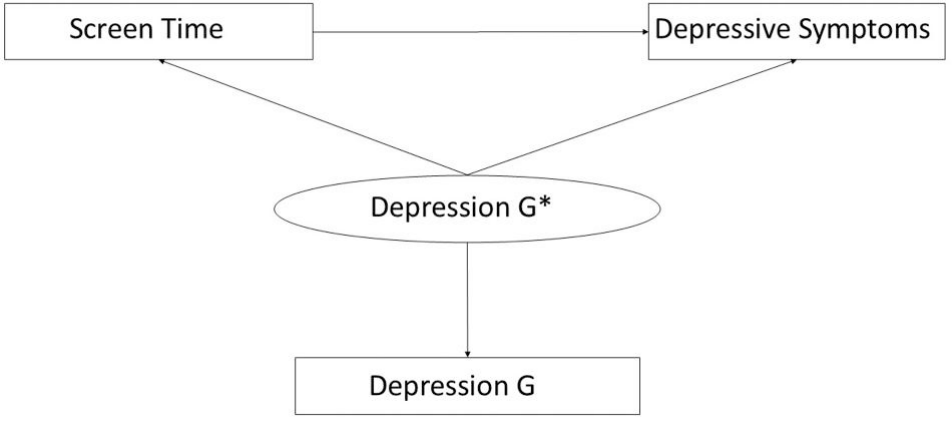
Gsens model. Square nodes represent observed variables and oval nodes represent latent variables. Depression G refers to observed PGSs for depression; Depression G* refers to latent PGSs that capture the heritability of depression.

Descriptive analyses were conducted. Associations between each type of screen time at ages 16, 22, and 26 years, and depressive symptoms at age 26 years were estimated by using linear regression. We examined the association between screen time and depressive symptoms by using an unadjusted model (Model 1), a model adjusted for sociodemographic factors (Model 2), and a model adjusted for both sociodemographic factors and PGSs for depression (Model 3). The Gsens was conducted by using the Gsens package (Model 4) [17, 18]. After adjusting for sociodemographic factors, we assessed the association between screen time and depressive symptoms accounting for a latent PGS capturing SNP-based heritability of depression in a structural equation model (Fig. 2). The genetic confounding effect is calculated as the decrease in this association after including the latent PGS. Additionally, where appropriate, we estimated the proportion of effect-size attenuation attributable to genetic confounding. The SNP heritability of depression at age 26 years was set at 0.05 in the Gsens model [38]. See Supplementary Section 2 for SNP-based heritability estimates and Supplementary Section 3 for the pseudo-R code.

We also conducted analyses to examine the associations between screen time at age 16 years and depressive symptoms at age 16 years (SNP-based heritability = 0.08) and the associations between screen time at ages 16 and 22 years and depressive symptoms at age 22 years (SNP-based heritability = 0.05). Sensitivity analyses were conducted using heritability values ranging from 0.01 to 0.10 and using PGSs constructed with a less stringent LD clumping threshold of *r*^2^ > 0.1. Additional analysis was conducted by using a binary measure of screen time. Each subtype of screen time was categorized into “>2 h per day” and “≤2 h per day” in line with previous studies reporting a nonlinear dose–response association between screen time and depression, with a cutoff at 2 hours per day [10, 39]. Complete-case analysis was also conducted as a sensitivity analysis.

## Results

### Descriptive statistics

Of 3003 participants, 1937 (64.5%) were females; most of them (85.9%) were from families with married parents; 1001 (33.3%) participants’ parent(s) had a degree; 77 reported NEET status at age 16 years (Table 1).

**Table 1:** Sociodemographic characteristics of participants (*N* = 3003).

	Proportions (%)
**Sex at birth**	
Female	1937 (64.5)
Male	1066 (35.5)
**Parental marital status**	
Married/remarried	2579 (85.9)
Never married	299 (10.0)
Divorced/separated/widowed	125 (4.2)
**Parental highest education level^a^**	
CSE/vocational	264 (8.8)
O-level	673 (22.4)
A-level	1065 (35.5)
Degree	1001 (33.3)
**Parental highest occupational social class^b^**	
I	195 (6.5)
II	808 (26.9)
III (non-manual)	779 (25.9)
III (manual)	635 (21.1)
IV	458 (15.2)
V	128 (4.3)
**NEET status^c^**	
Yes	77 (2.6)
No	2926 (97.4)

a CSE (Certificate of Secondary Education) and O-level were qualifications taken at age 16 years—now replaced by GCSEs (General Certificate of Secondary Education) in England, Wales, and Northern Ireland. A-levels are exams taken at age 18 years in these countries.

b Parental occupational social class was based on the higher of the mother or partner’s occupational social class by using the 1991 British Office of Population and Census Statistics classification.

c NEET status: not in education, employment, and training status at age 16 years.

The proportions of different types of screen time during weekdays and weekends are shown in Supplementary Table 2. At age 16 years, 34.2% of participants reported watching >2 hours of television, 41.8% reported using a computer for >2 hours, 27.1% reported texting for >2 hours, and 8.2% reported talking on a mobile phone for >2 hours during weekends. During weekdays, these proportions were lower for television and computer (24.0% and 29.7%) and similar for texting and talking on a mobile phone (21.5% and 5.6%). The proportions generally increased at age 22 years and even further at 26 years. The median SMFQ score was 4 [interquartile range (IQR): 2–9] at age 16 years, increased to 5 (IQR: 2–10) at age 22 years, and remained at 5 (IQR: 2–10) at age 26 years. Correlations for all screen-time measures are shown in Supplementary Table 8. Compared with participants excluded due to having <50% data on screen time or not having at least one assessment of depressive symptoms, those with ≥50% data on screen time and at least one assessment of depressive symptoms were more likely to be female, from married or widowed families, have parents with a higher education level, and be in NEET status (Supplementary Table 5).

### Associations between screen time and depressive symptoms

**Figure 3:**
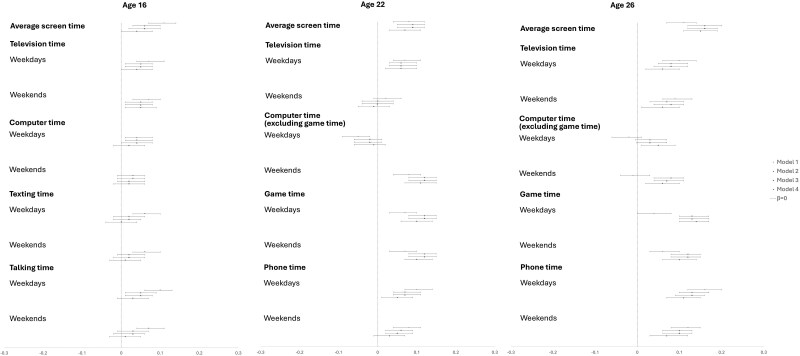
Associations between screen time at ages 16, 22, and 26 years, and depressive symptoms at age 26 years. Model 1: unadjusted model; Model 2: adjusted for sex, parental marital status, parental highest education level, parental highest occupational social classes, and NEET status; Model 3: adjusted for sex, parental marital status, parental highest education level, parental highest occupational social classes, NEET status, and PGSs for depression; Model 4: Gsens using Gsens model with latent PGSs for depression, adjusted for sex, parental marital status, parental highest education level, parental highest occupational social classes, and NEET status.

Table 2 shows the results of associations between each type of screen time at ages 16, 22, and 26 years, and SMFQ score at age 26 years. Unadjusted models (Model 1, Fig. 3) showed that some, but not all, types of screen time were associated with the SMFQ score. The average daily screen time at ages 16 years [β: 0.11, 95% confidence interval (CI) 0.07, 0.14], 22 years (β: 0.08, 95% CI 0.04, 0.12), and 26 years (β: 0.11, 95% CI 0.07, 0.14) was associated with depressive symptoms at age 26 years. In particular, time spent using a phone, tablet, or e-book at age 22 years (β: 0.10, 95% CI 0.07, 0.14 for weekdays; β: 0.08, 95% CI 0.04, 0.11 for weekends) and television time at age 26 years (β: 0.10, 95% CI 0.06, 0.14 for weekdays; β: 0.09, 95% CI 0.06, 0.13 for weekends) seem to be associated with depressive symptoms at age 26 years.

**Table 2:** Associations between screen time at ages 16, 22, and 26 years, and depressive symptom scores at age 26 years (*N* = 3003).

	Model 1^a^	Model 2^b^	Model 3^c^
	β (95% CI)	β (95% CI)	β (95% CI)
**Screen time at age 16 years (hours)**			
**Average daily screen time**	0.11 (0.07, 0.14)	0.06 (0.03, 0.10)	0.06 (0.02, 0.10)
**Weekdays**			
Television time	0.07 (0.04, 0.11)	0.05 (0.01, 0.08)	0.05 (0.01, 0.08)
Computer time	0.04 (0.01, 0.08)	0.04 (0.01, 0.08)	0.04 (0.01, 0.08)
Texting time	0.06 (0.03, 0.10)	0.02 (−0.02, 0.06)	0.02 (−0.02, 0.05)
Talking time	0.10 (0.06, 0.13)	0.05 (0.01, 0.09)	0.05 (0.01, 0.08)
**Weekends**			
Television time	0.07 (0.03, 0.10)	0.05 (0.01, 0.08)	0.05 (0.01, 0.08)
Computer time	0.03 (−0.01, 0.06)	0.03 (−0.01, 0.06)	0.02 (−0.01, 0.06)
Texting time	0.06 (0.03, 0.10)	0.02 (−0.01, 0.06)	0.02 (−0.02, 0.06)
Talking time	0.07 (0.04, 0.11)	0.03 (−0.01, 0.07)	0.03 (−0.02, 0.06)
**Screen time at age 22 years (hours)**			
**Average daily screen time**	0.08 (0.04, 0.12)	0.09 (0.05, 0.12)	0.09 (0.05, 0.12)
**Weekdays**			
Television time	0.07 (0.04, 0.11)	0.06 (0.03, 0.10)	0.06 (0.03, 0.10)
Computer time (excluding game time)	−0.05 (−0.09, −0.02)	−0.02 (−0.06, 0.01)	−0.02 (−0.06, 0.01)
Game time	0.07 (0.03, 0.10)	0.12 (0.08, 0.15)	0.12 (0.08, 0.15)
Phone time	0.10 (0.07, 0.14)	0.07 (0.04, 0.11)	0.07 (0.04, 0.11)
**Weekends**			
Television time	0.02 (−0.01, 0.06)	0.0002 (−0.04, 0.04)	−0.0006 (−0.04, 0.04)
Computer time (excluding game time)	0.08 (0.04, 0.11)	0.12 (0.08, 0.15)	0.12 (0.08, 0.15)
Game time	0.07 (0.03, 0.10)	0.12 (0.08, 0.15)	0.12 (0.08, 0.15)
Phone time	0.08 (0.04, 0.11)	0.06 (0.02, 0.09)	0.05 (0.02, 0.09)
**Screen time at age 26 years (hours)**			
**Average daily screen time**	0.11 (0.07, 0.14)	0.16 (0.12, 0.20)	0.16 (0.12, 0.19)
**Weekdays**			
Television time	0.10 (0.06, 0.14)	0.08 (0.05, 0.12)	0.08 (0.04, 0.12)
Computer time (excluding game time)	−0.02 (−0.06, 0.01)	0.03 (−0.004, 0.07)	0.03 (−0.002, 0.07)
Game time	0.04 (0, 0.08)	0.13 (0.10, 0.17)	0.13 (0.10, 0.17)
Phone time	0.16 (0.12, 0.20)	0.13 (0.10, 0.17)	0.13 (0.09, 0.16)
**Weekends**			
Television time	0.09 (0.06, 0.13)	0.07 (0.03, 0.11)	0.08 (0.04, 0.11)
Computer time (excluding game time)	−0.01 (−0.04, 0.03)	0.08 (0.04, 0.11)	0.07 (0.03, 0.11)
Game time	0.06 (0.03, 0.10)	0.12 (0.08, 0.15)	0.12 (0.08, 0.15)
Phone time	0.12 (0.08, 0.15)	0.10 (0.06, 0.13)	0.10 (0.06, 0.13)

a Unadjusted model.

b Adjusted for sex, parental marital status, parental highest education level, parental highest occupational social classes, and NEET status.

c Adjusted for sex, parental marital status, parental highest education level, parental highest occupational social classes, NEET status, and PGSs for depression.

After adjustment for sociodemographic confounders (Model 2, Fig. 3), including sex, parental marital status, parental highest education level, parental highest occupational social class, and NEET status, some existing associations between screen time and depressive symptoms in the unadjusted models changed. For example, the association with texting time at age 16 years (β: 0.02, 95% CI −0.02, 0.06 for weekdays; β: 0.02, 95% CI −0.01, 0.06 for weekends) attenuated. However, further adjustment for PGSs (Model 3, Fig. 3) did not attenuate the observed associations.

### Associations between screen time and depressive symptoms after adjustment for latent PGS scores capturing SNP heritability in depression

In general, most of the associations were further attenuated in the Gsens models that accounted for latent PGSs for depression (Model 4, Fig. 3 and Table 3). The association between average daily screen time at age 16 years and depressive symptoms at age 26 years were strongly attenuated (β = 0.04, 95% CI 0, 0.08, proportion attenuation = 42%), whereas the associations at ages 22 years (β = 0.07, 95% CI 0.03, 0.11) and 26 years (β = 0.15, 95% CI 0.11, 0.19) with depressive symptoms at age 26 years remained. The attenuation in the effect sizes with the Gsens ranged from 13% to 55% (mean = 30%). For example, the associations between time spent using a phone, tablet, or e-book at age 22 years and SMFQ scores at age 26 years were as follows for weekdays and weekends: β = 0.05, 95% CI 0.01, 0.09, 30%; β = 0.03, 95% CI −0.01, 0.07, 41%.

**Table 3:** Associations between screen time at ages 16, 22, and 26 years and depressive symptom scores at age 26 years in the Gsens model (Model 4) (*N* = 3003).

	Association^a^	Genetic confounding effect	Adjusted association^b^
	β (95% CI)	β (95% CI)	β (95% CI)
**Screen time at age 16 years (hours)**			
**Average screen time**	0.06 (0.03, 0.10)	0.03 (0, 0.05)	0.04 (0, 0.08)
**Weekdays**			
Television time	0.05 (0.01, 0.08)	0.01 (−0.01, 0.03)	0.04 (0, 0.08)
Computer time	0.04 (0.01, 0.08)	0.02 (0, 0.05)	0.02 (−0.02, 0.06)
Texting time	0.02 (−0.02, 0.06)	0.02 (0, 0.04)	0 (−0.04, 0.04)
Talking time	0.05 (0.01, 0.09)	0.02 (0, 0.05)	0.03 (−0.01, 0.07)
**Weekends**			
Television time	0.05 (0.01, 0.08)	0 (−0.02, 0.02)	0.05 (0.01, 0.09)
Computer time	0.03 (−0.01, 0.06)	0.01 (−0.01, 0.03)	0.02 (−0.02, 0.06)
Texting time	0.02 (−0.01, 0.06)	0.02 (0, 0.04)	0.01 (−0.03, 0.05)
Talking time	0.03 (0.01, 0.06)	0.02 (0, 0.04)	0.01 (−0.03, 0.05)
**Screen time at age 22 years (hours)**			
**Average screen time**	0.09 (0.05, 0.12)	0.02 (0, 0.04)	0.07 (0.03, 0.11)
**Weekdays**			
Television time	0.06 (0.03, 0.10)	0 (−0.01, 0.03)	0.06 (0.02, 0.10)
Computer time (excluding game time)	−0.03 (−0.06, 0.01)	−0.01 (−0.03, 0.01)	−0.01 (−0.06, 0.02)
Game time	0.12 (0.08, 0.15)	0.02 (0, 0.04)	0.10 (0.06, 0.14)
Phone time	0.07 (0.04, 0.11)	0.02 (0, 0.04)	0.05 (0.01, 0.09)
**Weekends**			
Television time	0 (−0.04, 0.04)	0.01 (−0.01, 0.03)	−0.01 (−0.05, 0.03)
Computer time (excluding game time)	0.12 (0.08, 0.15)	0 (−0.01, 0.03)	0.11 (0.07, 0.15)
Game time	0.12 (0.08, 0.15)	0.01 (−0.01, 0.03)	0.10 (0.07, 0.14)
Phone time	0.06 (0.02, 0.09)	0.02 (0, 0.04)	0.03 (−0.01, 0.07)
**Screen time at age 26 years (hours)**			
**Average screen time**	0.16 (0.12, 0.20)	0.01 (−0.01, 0.03)	0.15 (0.11, 0.19)
**Weekdays**			
Television time	0.08 (0.05, 0.12)	0.02 (0, 0.04)	0.06 (0.02, 0.10)
Computer time (excluding game time)	0.03 (0, 0.07)	−0.02 (−0.04, 0)	0.05 (0.01, 0.09)
Game time	0.13 (0.10, 0.17)	0 (−0.02, 0.02)	0.14 (0.10, 0.17)
Phone time	0.13 (0.10, 0.17)	0.02 (0, 0.04)	0.11 (0.07, 0.15)
**Weekends**			
Television time	0.08 (0.04, 0.11)	0.02 (0, 0.04)	0.06 (0.01, 0.10)
Computer time (excluding game time)	0.07 (0.03, 0.10)	0.01 (−0.01, 0.03)	0.06 (0.02, 0.10)
Game time	0.12 (0.08, 0.15)	0.02 (0, 0.04)	0.10 (0.06, 0.14)
Phone time	0.10 (0.06, 0.13)	0.02 (0, 0.04)	0.07 (0.03, 0.12)

a Adjusted for sex, parental marital status, parental highest education level, parental highest occupational social classes, and NEET status.

b Additionally adjusted for latent PGSs for depression in Gsens model.

The effect sizes of genetic confounding in the associations between screen time and outcomes at ages 16 and 22 years were similar to those for the outcome at age 26 years. There was evidence of genetic confounding for the associations between average screen time at age 16 years and depressive symptoms at ages 16 years (β = 0.13, 95% CI 0.08, 0.17, proportion attenuation = 25%) and 22 years (β = 0.01, 95% CI −0.03, 0.06, proportion attenuation = 69%), and there was no evidence of genetic confounding in the association between screen time at age 22 years and depressive symptoms at age 22 years (Supplementary Table 9).

Genetic confounding was similar across heritability values ranging from 0.01 to 0.10, with greater levels observed at higher heritability parameters (Supplementary Table 10). The results were not substantially changed when the PGS derived with an LD clumping threshold of *r*^2^ > 0.1 was used. Additional genetic confounding was observed in the associations between average screen time at age 22 years and depressive symptoms at age 26 years (Supplementary Table 11). Attenuation in the associations between >2 hours of different types of screen time and depressive symptoms at age 26 years was also observed in the genetic sensitivity models (Supplementary Table 12). Results from the complete-case analyses are presented in Supplementary Table 13.

## Discussion

Based on the large longitudinal dataset, we found that some—but not all—measures of screen time were associated with higher levels of depressive symptoms. In particular, the average daily screen time at age 16 years, time spent using a phone, tablet, or e-book at age 22 years, and television time at age 26 years were associated with depressive symptoms at age 26 years. However, these associations between screen time and depressive symptoms were attenuated after accounting for genetic confounding. Due to the complex nature of genetic architecture, PGSs captured only a small proportion of the total heritability [15] and were insufficient to account for total genetic confounding. Associations between screen time and depressive symptoms persisted when PGSs were included as covariates, but were extensively attenuated when latent PGSs that incorporate information about SNP-based heritability were used. We adjusted for ancestry principal components to minimize the impact of population stratification. Nevertheless, results of the Gsens analysis might reflect several sources of genetic confounding. Besides confounding from direct genetic effects [18], this may include the impact of SNPs in LD with included SNPs and confounding due to family-level processes of assortative mating and genetic nurture [40].

Studies have suggested that genes played a role in both screen time [11] and mental health [41–43], providing potential for genetic confounding. However, most previous observational studies of mental health outcomes related to screen time [44, 45] have not considered genetic confounding. Of the two studies that have investigated genetic confounding by using the Gsens analysis, one focused on screen time and psychiatric problems in children based on 1-year follow-up data [20] and the other focused on media use and mental health measured at the same time point in early adulthood [19]. Both reached a similar conclusion to this paper, i.e. that genetic confounding explained most of the association between screen time and mental health [19, 20]. A PGS for depression could be associated with screen time if screen time is a consequence of, rather than a cause of, depression, or if the genetic variants affect both screen time and depression through pleiotropic pathways [11].

In this study, using a large UK longitudinal dataset with three waves of data collected during adolescence and early adulthood, we explored the associations between different types of screen time and later depressive symptoms. We investigated these associations by using information on exposures and outcomes across multiple ages and applied various approaches to interrogate genetic confounding. However, several limitations should be considered. We used self-reported screen time, which can lead to recall bias and social desirability bias [46]. The collected screen-time data were ordinal variables rather than continuous. However, we included all prospectively collected screen-time data in ALSPAC and calculated the average screen time to test the robustness of our findings across different types of screen time and at multiple time points for the same individuals. Additionally, we focused only on White participants due to the available genetic data in ALSPAC. Trans-ancestry GWASs are needed and data on screen time, mental health, and genetic information among populations of non-European ancestry will be helpful to triangulate our findings.

## Conclusions

We found that genetic confounding is likely to partly explain the association between screen time and depressive symptoms in adolescents and young adults. There were weaker associations between different types of excessive screen time on weekdays or weekends and depressive symptoms among adolescents and young adults after accounting for genetic confounding.

## Supplementary Material

dyag079_Supplementary_Data

## Data Availability

The informed consent obtained from ALSPAC participants does not allow the data to be made available through any third-party-maintained public repository. Supporting data are available from ALSPAC upon request under the approved proposal number B4380. Full instructions for applying for data access can be found at http://www.bristol.ac.uk/alspac/researchers/access/. The ALSPAC study website contains details of all available data (http://www.bristol.ac.uk/alspac/researchers/our-data/).
