# Supplementary material for: Exploring genetic confounding of the associations between screen time and depressive symptoms in adolescence and early adulthood
Source: Int J Epidemiol. 2026 Jun 11;55(3):dyag079. doi: 10.1093/ije/dyag079 (PMC13260654; doi:10.1093/ije/dyag079)
Supplement: dyag079_Supplementary_Data [file dyag079_supplementary_data.zip › ije-2025-02-0324-File006.docx]

**Supplementary Section 1**

Study data were collected and managed using REDCap electronic data capture tools hosted at the University of Bristol [1]. REDCap (Research Electronic Data Capture) is a secure, web-based software platform designed to support data capture for research studies. Please note that the [study website](http://www.bristol.ac.uk/alspac/researchers/our-data/) contains details of all the data that is available through a fully searchable data dictionary and variable search tool. Samples were removed if they had discordant sex information, outlying heterozygosity, a high proportion of individual missingness, or due to relatedness or non-European population ancestry. After quality control procedures, removing those with cryptic relatedness greater than 0.05 or who had withdrawn consent, 2,056 of the 9,912 genotyped participants (using Illumina HumanHap500-quad genotyping array) were excluded [2, 3].

**Supplementary Section 2**

The single nucleotide polymorphism (SNP)-based heritability estimates of the child-reported Short Mood and Feelings Questionnaire (SMFQ) for depressive symptoms in ALSPAC at age 16.5 years, 18 years, and 18.5 years were 8%, 3%, and 5%, respectively, and remained relatively stable after age 20, as estimated using Genome-wide Complex Trait Analysis (GCTA) software [4].

**Supplementary Section 3**

Followings are R codes for models:

#Model 1

M1 <- lm (MH ~ ST)

#Model 2

M2 <- lm (MH ~ ST+SEX+MARR+PEDU+PCLASS+NEET)

#Model 3

M3 <- lm (MH ~ ST+SEX+MARR+PEDU+PCLASS+NEET+MDD)

#Model 4

# rxy = the observed phenotypic correlation between exposure X and outcome Y

M2 <- lm (MH ~ ST+SEX+MARR+PEDU+PCLASS+NEET)

r_xy<-coefficientr(M2)

#rgx the observed correlation between phenotype X and the observed polygenic score for depression

t1<-cor.test(ST, MDD, method = "pearson")

r_gx<-t1$estimate

# rgy = the observed correlation between phenotype Y and the observed polygenic score for depression

t2<-cor.test(MH, MDD, method = "pearson")

r_gy<-t2$estimate

M4<-gsensY_prop(rxy= r_xy,

rgx = r_gx,

rgy = r_gy,

n=N,

h2=H2)

Notes:

ST refers to a specific type of screen time

MH refers to Short Mood and Feelings Questionnaire (SMFQ) scores

SEX refers to participants’ sex

MARR refers to parental marital status

PCLASS refers to parental highest occupational social classes

NEET refers to not in education, employment and training (NEET) status

MDD refers to polygenic scores for depression

H2 refers to SNP-based heritability estimates

correlationr refers to a function that converts standardized mean difference into correlation and provides 95% confidence intervalsFor more information, please refer to <https://github.com/JiayaoXu2023/Gsens_ST-MH>

**References**

1. Harris, P.A., et al., *Research electronic data capture (REDCap)--a metadata-driven methodology and workflow process for providing translational research informatics support.* J Biomed Inform, 2009. **42**(2): p. 377-81.

2. Riglin, L., et al., *Early manifestations of genetic liability for ADHD, autism and schizophrenia at ages 18 and 24 months.* JCPP Adv, 2022. **2**(3).

3. Fraser, A., et al., *Cohort Profile: the Avon Longitudinal Study of Parents and Children: ALSPAC mothers cohort.* Int J Epidemiol, 2013. **42**(1): p. 97-110.

4. Sallis, H., et al., *Genetics of depressive symptoms in adolescence.* BMC Psychiatry, 2017. **17**(1): p. 321.
